# Health beliefs of unmarried adult Saudi individuals toward safe marriage and the role of premarital screening in avoiding consanguinity: a nationwide cross-sectional study

**DOI:** 10.3389/fpubh.2024.1379326

**Published:** 2024-06-19

**Authors:** Reem M. Alwhaibi, Afrah K. Almuwais, Madawi Alotaibi, Hanan M. AlTaleb, Shatha M. Alsamiri, Ruqaiyah Khan

**Affiliations:** ^1^Department of Rehabilitation Sciences, College of Health and Rehabilitation Sciences, Princess Nourah Bint Abdulrahman University, Riyadh, Saudi Arabia; ^2^Department of Basic Health Sciences, Deanship of Preparatory Year for the Health Colleges, Princess Nourah Bint Abdulrahman University, Riyadh, Saudi Arabia

**Keywords:** premarital screening, susceptibility, benefits to action, consanguineous marriage, safe marriage, genetic counseling, barriers to action, Saudi Arabia

## Abstract

**Introduction:**

Premarital screening (PMS) is an essential global measure that seeks to reduce the occurrence of specific genetic disorders and sexually transmitted diseases common in consanguineous marriages. Due to the lack of a nationwide study, this research was designed to comprehend how unmarried individuals perceive the risks and benefits of PMS.

**Method:**

A cross-sectional study was conducted using an online questionnaire distributed through different social media platforms, responses from the native adult population (18–49 years) Saudi Arabia was only included in the study. The questionnaire was based on the Health Belief Model (HBM) to assessing seven different constructs including susceptibility, seriousness, benefits-, barriers-, & cues- to action, self-efficacy, and social acceptance. Data frequency was represented by mean and standard deviation; chi-square and *t*-tests were conducted for the comparison of independent and dependent variables. A multinomial logistic regression was used to predict factors influencing decisions related to PMS.

**Results:**

1,522 participants completed the survey, mostly 18–25 years old and most of them were women. The majority were single with 85 men and 1,370 women. Most participants (59.6%) believed their parents were related, while 40.5% did not. 122 respondents reported they had to marry within their tribe. Findings revealed significant correlations among all HBM themes, with varying strengths. Notably, a moderate positive relationship was found between the perception of benefits and cues to action, suggesting that enhancing the perceived benefits of PMS could facilitate safe marriage practices. Multinomial regression analysis revealed that demographic factors and health beliefs significantly influence individuals’ intentions and behaviors toward PMS and safe marriage.

**Conclusion:**

The study concludes that by identifying and addressing barriers, and promoting positive social acceptance, PMS can significantly contribute to preventing genetic diseases and promoting safe marriage practices, although the cross-sectional design limits the establishment of causal relationships and further research is needed.

## 1 Introduction

Consanguineous marriage (CM) refers to the union between individuals who share a close blood relationship, such as cousins or other closely related relatives (1, 2). It is widespread in numerous cultures worldwide, encompassing specific areas in the Middle East, Asia, and Africa. Individuals who partake in consanguineous matrimony intend to safeguard cultural customs, uphold familial connections, reinforce social relationships, or guarantee the transmission and conservation of wealth within the kinship (3). Nevertheless, CM also elevate the probability of acquiring genetic disorders and can have ramifications for the well-being of progeny, such as an augmented propensity for congenital abnormalities and hereditary ailments (4, 5).

The prevalence of CM varies across the globe, for instance, in some communities of North Africa, the Middle East, and West Asia, intra-familial unions collectively account for 20–50% or more of all marriages (4). Globally 8.5% of children have consanguineous parents, and approximately 20% of the human population lives in communities practicing endogamy (6). In Western and European countries, the prevalence of CM does not surpass 0.5% but in gulf countries it ranges between 40 and 60% (5, 7). For instance, in Qatar, the rate of consanguinity is approximately 54%, with first cousins’ marriages being the most prevalent (5). A study revealed remarkably high occurrence of consanguineous marriage in Oman (8), with over half (52%) of marriages being consanguineous. The prevalence of CM in Saudi Arabia ranges between 42 and 67%, with varying estimates across different cities. The prevalence of CM in cities like Mecca, Madinah, and Riyadh varies between 40 and 67%. These statistics emphasize the substantial occurrence of CM in specific cultures and the influence it exerts on population dynamics and genetic health results (1).

The high incidence of CM is a pivotal contributor to the transmission of inherited hemoglobinopathies (9). Hemoglobinopathies are a group of inherited blood disorders characterized by abnormalities in the structure or production of hemoglobin, resulting in impaired oxygen transport and potential complications such as anemia, organ damage, and chronic pain (10, 11). Sickle cell disease (SCD) and thalassemia, the most common hemoglobinopathies, require genetic counseling, regular blood transfusions, and potentially curative treatments like bone marrow transplantation and gene therapy (12).

SCD is a genetic disorder with high morbidity and mortality rates (12). According to the Ministry of Health (MOH) in 2019, the prevalence of SCD in Saudi Arabia was estimated to be 0.26% of the affected individuals and 4.2% were carriers of the sickle-cell trait, with the Eastern province having the highest prevalence (17% carriers) and 1.2% affected individuals (13). Thalassemia is a common hereditary blood disease in Saudi Arabia with an annual incidence rate of 0.05%, leading to a lack of hemoglobin and red blood cells in the body (14).

Such disorders may be mitigated by the implementation of the Premarital Screening Program (PMS). The Saudi MOH defined PMS as a medical examination conducted by individuals planning to exclude any possible conditions that can be passed to their offspring. This includes infectious and genetic blood disorders such as sickle cell anemia, thalassemia, hepatitis B, hepatitis C, and HIV/AIDS (15). This measure is also expected to alleviate the economic burden encountered by individuals and the government arising from having children with these conditions (16). Participation in premarital screening programs is voluntary in most countries. However, it has been made compulsory in others, including Saudi Arabia, in 2004 (9).

Although good-to-fair levels of knowledge about PMS have been reported by multiple studies (17–24), the knowledge of the complications caused by CM were not given significant emphasis in the current literature. Nevertheless, studies have shown that participants’ answers were incorrect regarding the symptoms of paralysis as a complication of CM (25). Higher levels of knowledge were associated with a positive family history of genetic diseases, high family income, and education (26, 27).

Additionally, many people reaching up to 90%, still choose to get married despite the incompatible results (26). The reasoning behind their decision was due to their inability to cancel their plans for the wedding, emotions toward their partner, lack of awareness, religious reasons, and social stigma (21, 26).

A way of looking into this behavior is using the Health Belief Model (HBM), a psychological and behavioral theory frequently employed to interpret one’s behavior (28). The HBM helps understand why people do not implement disease prevention strategies (28). It views attitude as a way a person perceives a certain behavior positively or negatively, which is dependent upon its consequences (25). Our thorough literature survey concluded that there is a dearth of studies related to PMS and its association with beliefs, attitudes, barriers, social acceptability, and cues to action. Therefore, this study was undertaken to investigate the health beliefs of unmarried individuals toward safe marriage and the role of PMS in preventing genetic diseases erupting from CM.

## 2 Materials and methods

### 2.1 Study design

A cross-sectional survey was conducted using a structured, self-reported online questionnaire completed by the participants. The survey was distributed via social media platforms between 7 August 2023 and 8 September 2023.

### 2.2 Participants and sample size

Male and female participants were invited to participate in the survey using the following criteria. Native adult population (between the age of 18–49 years) from all provinces in Saudi Arabia was the target population. A convenience sampling method was used in this study. The estimated sample size was 384 based on a population of 7,257,821 unmarried individuals within this age range, ensuring a 5% margin of error and 95% confidence level.

#### 2.2.1 Inclusion criteria

All the responses from unmarried participants between 18 and 49 years of age who were citizens or residents of Saudi Arabia and understood and read English or Arabic language were included in the study. Only the responses of the participants who answered the questionnaire completely were included.

#### 2.2.2 Exclusion criteria

Any response from participants who were currently married or were aged below 18 or above 49 years of age, were non-residents, were not citizens of Saudi Arabia, or did not understand and read English or Arabic language were excluded. In addition, the responses of those who did not provide consent to participate in this study were not included.

### 2.3 Measurement tool

**Figure 1:**
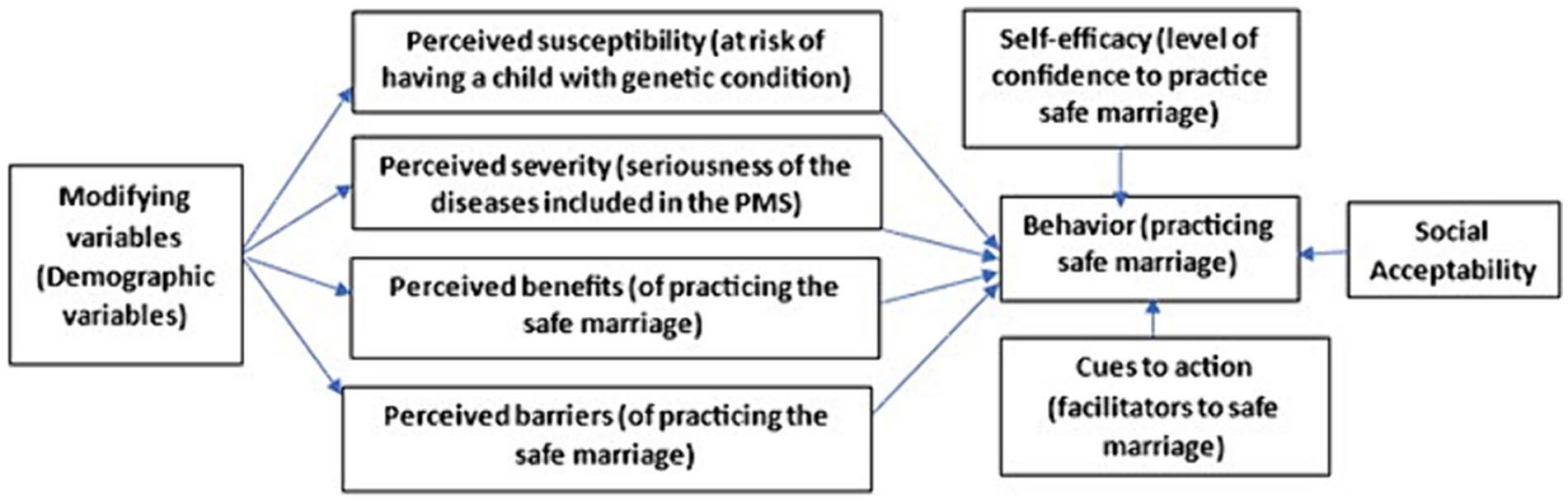
HBM constructs for different themes included in this study.

The online questionnaire was designed based on the Health Belief Model (HBM), as shown in Figure 1. It included a total of 45 questions across eight parts: demographics, perceived susceptibility, perceived severity, perceived benefits, perceived barriers, cues to action, self-efficacy, and social acceptability. Questions in all parts except demographics were measured on a five-point Likert scale, with one representing “strongly disagree” and five representing “strongly agree.” Except for the perceived barriers, which were reverse coded to clearly exhibit the extremities of barriers.

The questionnaire was made online using Google Forms. Google Forms is a survey management software that is included as a component of the free, online Google Docs Editors suite provided by Google. This online questionnaire was spread over nine different screens taking approximately 20 min to complete. The first screen provided the introduction to the study and took the participants’ consent followed by a second screen for demographic information. Seven other screens followed the second screen corresponding to the seven themes discussed as follows.

#### 2.3.1 Perceived susceptibility

It refers to the degree to which an unmarried couple perceives the risk of incompatible marriages. This was measured using a 5-point Likert scale consisting of five items, with scores ranging from 5 to 25. Higher scores indicate a higher perceived susceptibility to having a child with a genetic condition.

#### 2.3.2 Perceived seriousness

This is defined as an unmarried couple’s perception of the seriousness of the diseases covered by PMS, including sickle cell anemia, Thalassemia, Hepatitis, and acquired immunodeficiency syndrome (AIDS). It also measures the difficulties that could arise from these conditions, such as physical and financial burden, family issues, and susceptibility to future illnesses. This was measured using a 5-point Likert scale consisting of five items, and the average of all the questions was considered for comparison. A higher mean score indicated a higher perceived severity toward having a child with a genetic condition.

#### 2.3.3 Perceived benefit

This refers to how unmarried couples view the advantages of engaging in safe marriage practices that would minimize the chances of having a child with a genetic condition. This was measured using a five-item questionnaire with a five-point Likert scale, and the average of all the questions was considered for comparison. A higher mean score indicated a greater perceived benefit of following recommended preventive marriage behaviors.

#### 2.3.4 Perceived barriers

These refer to the factors that prevent individuals from engaging in safe marriage practices. For example, canceling a marriage may be difficult, inconvenient, and result in social problems. These factors may discourage someone from taking the desired action of practicing safe marriage. A seven-item questionnaire was used to measure perceived barriers, using a five-point Likert scale. The average of all the questions was considered for comparison, with a higher mean score indicating a greater perceived barrier to canceling marriage.

#### 2.3.5 Cues to action

This refers to factors that can help promote a safe marriage. These cues can be either internal (e.g., having a disability) or external (e.g., MOH educational campaigns, advice from friends, and the illness or disability of a family member). A Likert scale consisting of four items was used to measure the impact of these cues, and the average of all the questions was considered for comparison. A higher score indicated a greater influence of cues to encourage practicing safe marriage.

#### 2.3.6 Self-efficacy

This is the level of confidence that unmarried couples have in being able to cancel their marriage if the results are incompatible. This was measured using a Likert scale of three items, and the average of all the questions was considered for comparison. A higher score indicated that the individual has more confidence in following the recommended behavior of having a safe marriage.

#### 2.3.7 Social acceptability

Although not a direct factor in the health belief model, the perception of social acceptance plays a crucial role in determining the implementation of or resistance to safe marriage practices. This measure assesses how unmarried individuals view safe marriage as socially acceptable within their social networks, including family, friends, and the community. The assessment was conducted using a Likert scale comprising three items, and the average of all the questions was considered for comparison. A higher score showed that the person has greater social approval for adhering to the suggested practice of having a safe marriage.

### 2.4 Questionnaire translation

Since, the questionnaire was developed in English, it was translated to Arabic and then reverse translated from Arabic to English with the help of language experts available at the Princess Nourah University. The translation process involved multiple steps to ensure the accuracy, equivalence, and understandability of the translated questionnaire. Firstly, the questionnaire was professionally translated by a native speaker fluent in both the source and target languages. Next, a back-translation process was conducted by another bilingual expert to ensure the accuracy and consistency of the translated version. Finally, the translated version of the questionnaire was reviewed by a panel of experts, including bilingual individuals and the authors, to assess its clarity and suitability for the target population.

### 2.5 Validity and reliability of the questionnaire

A group of six experts from PNU reviewed the questionnaire to assess its content validity and clarity. Subsequently, a pre-test was conducted on 20 singles to ensure face validity. If any items in the questionnaire were found to be unclear, they were modified to ensure that all participants could understand the questions correctly. Finally, the internal consistency of the questionnaire was tested using Cronbach’s alpha test, which is calculated as a function of the number of test items and the average inter-correlation among the items. Alpha value provides insights into how closely related a set of test items are as a group, helping in assessing the reliability of their measurements.

### 2.6 Data collection

The link to online questionnaire was shared through different social media platforms like Twitter and WhatsApp. Since, Twitter is one of the major social platforms used by the youth across the globe, we targeted it to maximize our reach to the required sample population. The data was collected without any identifier of the participants; however, each response was saved with a unique response ID for easy differentiation. The data was secured with a password and was only accessible to the authors of this study.

Google forms does not record responses if the participant did not complete the questions marked as required, hence only fully completed responses were included for analyses in this survey. Additionally, Google forms provides option to go back and review the previous questions to ensure accuracy of the answers. The participants were also allowed to have a final review and save their responses. However, once the submission was complete the responses were inaccessible to the participants for any other modification. Google forms uses cookies and IP addresses to identify and differentiate unique responders, also we used “one response per participant” which requires signing in to prevent duplication of responses from the same participant. Total completed responses were 1,522 out of 1,673 participants who gave their consent, so the completion rate for our survey was 90.97%.

### 2.7 Statistical analysis

Pearson’s correlation coefficient (PCC) was used to measure the correlation between different themes used in this study, which helped to understand the direction (+/−) and strength of a linear relationship. PCC ranges from −1 to 1; where −1 = perfect negative correlation, 1 = perfect positive correlation and 0 = no correlation. PCC also helped in determining if there was any statistically significant relationship between the variables. Descriptive statistics and the chi-square test were used to summarize data and determine associations between variables.

One-way ANOVA (ANalysis Of VAriance) calculates the variation within (more than three) groups and between groups to assess whether the differences are by chance or if they are meaningful. Since, there were more than three themes in our study, ANOVA was carried out to determine whether there are any statistically significant differences between the means of different themes. After performing the ANOVA, a post-hoc test (Tukey’s test) was employed to determine which specific theme means are significantly different from one another. It corrects for multiple comparisons and allows to identify pairwise differences between groups.

Multiple regression is used to explore the relationship between a dependent and multiple independent variables. It helps in determining the extent to which the independent variables predict the dependent variable and assesses the significance of each predictor. It helps to understand the impact of multiple predictors on the outcome variable and can be used for prediction or hypothesis testing. Our study used multiple regression to understand the dependence of one theme against the others. Moreover, a multiple logistic regression was employed to predict the factors that impact a single’s decision regarding a safe marriage, given that their PMS results are incompatible. A statistical significance was set at *p* < 0.05.

## 3 Results

### 3.1 Reliability test of the questionnaire

The overall Cronbach’s alpha value for all the questions was found to be 0.757, hence the internal consistency of the questionnaire used for the PMS of Saudi Population was acceptable.

### 3.2 Correlation between the themes of PMS questionnaire

Pearson’s correlation between all the themes was estimated and it was found that all the associations were significant, but its strength varied between moderate to very low. As evident from Table 1, most of the relationships were positive except for the Barriers to action which was negatively associated to all the themes. Benefits to action had a significant moderate positive relationship with cues to action, which may imply that if the perception of benefitting from PMS is improved it will facilitate safe marriage.

**Table 1:** The correlations between the themes.

		Perceived susceptibility	Perceived seriousness	Benefits to action	Barriers to action	Cues to action	Self-efficacy	Social acceptance
Perceived susceptibility	*r*	1						
Perceived seriousness	*r*	0.338^**^	1					
	*p*	0.00						
Benefits to action	*r*	0.300^**^	0.384^**^	1				
	*p*	0.00	0.00					
Barriers to action	*r*	0.025	−0.102^**^	−0.268^**^	1			
	*p*	0.339	0.003	0.00				
Cues to action	*r*	0.285^**^	0.293^**^	0.472^**^	−0.178^**^	1		
	*p*	0.00	0.00	0.00	0.00			
Self-efficacy	*r*	0.127^**^	0.214^**^	0.373^**^	−0.311^**^	0.363^**^	1	
	*p*	0.00	0.00	0.00	0.00	0.00		
Social acceptance	*r*	0.056^*^	0.139^**^	0.332^**^	−0.291^**^	0.286^**^	0.467^**^	1
	*p*	0.029	0.00	0.00	0.00	0.00	0.00	

^**^Correlation is significant at the 0.01 level (2-tailed).

^*^Correlation is significant at the 0.05 level (2-tailed).

*p* = statistical significance.

*r* = Pearson correlation coefficient.

### 3.3 Descriptive statistics

A total of 1,522 individuals participated in this cross-sectional study, predominantly aged 18–25, with a higher number of females than males. Most participants are single or unmarried, with 85 males and 1,370 females. Since, only a smaller number of males participated as compared to the females, the outcomes of our study are more specific to females. Geographically, the central region (1099) had the highest representation while the north region (42) had the lowest. Educationally, most participants had a university degree (906) followed by higher schoolers (420). Majority of the responders were students (1181), followed by government sector employees (143) and private sector employees (100). In terms of family income, the majority reported it to be above 10,000 SAR/month. 7.09% of the participants were diagnosed with a genetic disease, and 17.27% of the responders reported their 1st degree relative to have been diagnosed with a genetic condition. Majority of the participants (59.6%) agreed that their parents were somehow related whereas 40.5% denied any kind of relationship between their parents. 122 participants agreed that they were obliged to marry among the relatives or tribe (Table 2).

**Table 2:** Sociodemographic data with respect to Gender (*n* = 1,522).

Variables		Total	Males	Females
Age (years old)	18–25	1,286	46	1,240
	26–33	154	32	122
	34–41	11	56	67
	42–49	4	11	15
Marital status	Single or unmarried	1,455	85	1,370
	Engaged or planning to marry	67	8	59
Location	North region	52	10	42
	South region	168	13	155
	East region	54	3	51
	West region	89	7	82
	Central region	1,159	60	1,099
Education	High School and below	420	16	404
	Diploma	101	6	95
	University	906	65	841
	Postgraduate	95	6	89
Employment status	Unemployed (not a student)	94	3	91
	Student	1,181	42	1,139
	Government sector	143	29	114
	Military sector	4	3	1
	Private sector	100	16	84
Family income	<10,000 SAR/month	524	36	488
	>10,000 SAR/month	998	57	941
Have you been diagnosed with genetic disease?	Yes	108	2	106
	No	1,414	91	1,323
Have you ever been married?	Yes	41	4	37
	No	1,481	89	1,392
Do have any of the following disability?	No disability	1,494	91	1,403
	Mental/learning disability	4	0	4
	Visual/hearing disability	20	1	19
	Physical disability	4	1	3
Have any of your 1st-degree family members (parents or siblings) been diagnosed with a genetic disease?	Yes	263	11	252
	No	1,019	68	951
	Do not know	240	14	226
Does any of your 1st-degree family members (parents or siblings) have any of the following disabilities?	No disability	1,395	86	1,309
	Mental/learning disability	56	4	52
	Visual/hearing disability	34	1	33
	Physical disability	37	2	35
Which of the following best describes the relationship between your parents?	No relationship	616	37	579
	1st degree cousin	335	21	314
	2nd degree cousin	204	10	194
	related to the same tribe	367	25	342
Do the traditions of your family, tribe, or society oblige you to marry from your relatives?	Yes	122	8	114
	No	1,253	80	1,173
	Do not know	147	5	142

### 3.4 Difference between the mean of the themes with respect to (wrt) different age groups

**Figure 2:**
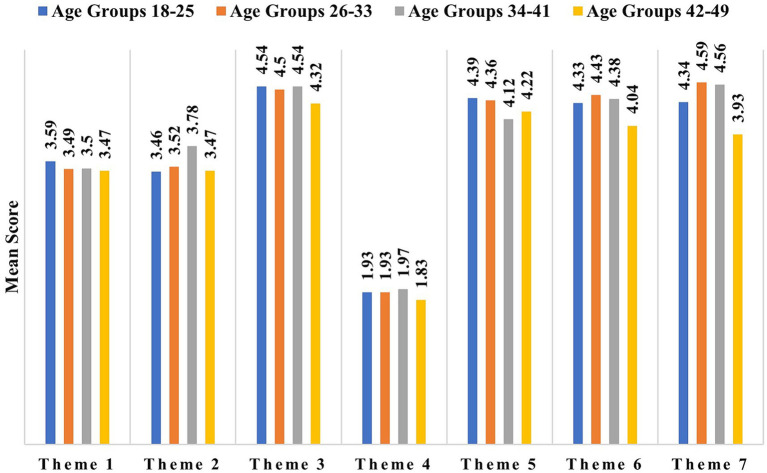
The mean score of different themes according to age groups, where Theme 1 to Theme 7 correspond sequentially to Perceived susceptibility, Perceived seriousness, Benefits to action, Barriers to action, Cues to action, Self-efficacy, and social acceptance, respectively.

Perceived susceptibility was highest among the 18–25 age group (3.59 ± 0.67) and lowest among 42–49 group (3.47 ± 1.24). Perceived seriousness was highest among the 34–41 age group (3.78 ± 1.07). The mean of Benefits to action was almost the same for all the age groups (~4.5) except for 42–49 (4.32 ± 1.08). Similarly, Barriers to action (~1.9) were the same for all age groups except for 42–49 (1.83 ± 0.96). Cues to action theme had a similar mean for responders under 34 years of age, whereas it was lowest in the 34–41 age group. Self-efficacy had the poorest mean in the oldest age group, but it was highest among 26–33-year-old participants. Similarly, the mean of social acceptance was lowest among participants above 42 years and highest among participants between 26 and 33 years (Figure 2). Female participants (3.59 ± 0.70) are found to be slightly more susceptible than males (3.42 ± 0.77). Similarly, seriousness (3.57 ± 0.86), benefits to action, cues to action, self-efficacy and social acceptance for PMS were more common among females than males, except for Barriers to action (Supplementary Tables 1A,B). All these variations in the means of themes cannot be based merely on gender differences, it may also be due to low number of male participants in the presented study.

Outcomes of ANOVA followed by post-hoc Tukey’s test for variances of the mean of all themes among age groups showed that perceived susceptibility has no significant variance between different age groups. Seriousness was found to have significant variation between the 18–25 and 34–41 year age group (*p* = 0.029). Similarly, the mean of cues to action was noticeably varying between the 18–25 and 34–41 year age group (*p* < 0.042). Additionally, the mean of social acceptance was found to vary significantly (*p* = 0.003) between the 18–25 and 26–33 years age categories. It was also found to be significantly different between the 42–49 years age category and 26–33 years (*p* = 0.023) and 34–41 years (*p* = 0.047).

### 3.5 Difference in the means of the themes for previously married and unmarried participants

Respondents who were previously married had higher mean for every theme than those who were not married, except for the Cue to action, where those who were married (4.30 ± 0.96) had lower mean than those who were never married (4.37 ± 0.79). *t*-test for independent samples test value (*t* = −1.880; df = 1,520; CI = −0.52 to 0.11) showed that the mean scores between these two groups were significantly different (*p* = 0.060). No other significant differences were noted in any other themes (Supplementary Tables 2A,B).

### 3.6 Difference in the means of responders with different education status

The mean score of “Barriers to action” theme was found to be significantly lower (*p* < 0.05) for the Diploma holders (3.75 ± 0.74) when compared to those educated up to High School (4.03 ± 0.74), or University (4.11 ± 0.77), or Postgraduate (4.20 ± 0.70). Similarly, those who were qualified with a PG degree (4.62 ± 0.63) had the highest social acceptability, which varied significantly (*p* = 0.002) against diploma holders (4.17 ± 0.10) who reported the lowest social acceptability. An ascending mean score of the social acceptance theme was noticed for those who had a diploma or a university degree (4.39 ± 0.84) (*p* = 0.008) (Supplementary Tables 3A,B).

### 3.7 Difference in the mean of the themes wrt to the income level

Responders who had a family income of less than 10 k (4.31 ± 0.91) showed less social acceptability than those whose family income was more than 10 k (4.341 ± 0.83). *t*-test for independent samples test value (*t* = −2.063; df = 1,520; 95%CI = −0.18 to −0.01) showed that the mean scores between these two groups were significantly different (*p* = 0.039). No other significant difference was noted in any other theme (Supplementary Table 4).

### 3.8 Difference in the mean of responders diagnosed with genetic disease

Responders diagnosed with any type of genetic disorder (3.94 ± 0.76) showed more susceptibility toward PMS than those who were not (3.55 ± 0.69). *t*-test for independent samples test value (*t* = −5.632; df = 1,520; 95%CI = −0.53 to −0.26) showed that the mean scores between these two groups were significantly different (*p* = 0.000). Contrastingly, those who were diagnosed with any genetic disorder found that “Cues to action” were less beneficial (4.22 ± 0.87) than those who did not report any genetic disorder (4.38 ± 0.79). The *t*-test showed that mean scores for these two groups were significantly (*p* = 0.044) different (*t* = 2.016; df = 1,520; 95%CI = −0.04 to −0.32). No other significant difference was noted in any other theme (Supplementary Table 5).

### 3.9 Variation in mean of themes when 1st degree relative had genetic anomalies

Responders whose 1st-degree members were diagnosed with any type of genetic disorder (3.81 ± 0.73) showed more susceptibility toward PMS than those who were not (3.50 ± 0.69). *t*-test for independent samples test value (*t* = −6.538; df = 1,580; 95%CI = −0.41 to −0.22) showed that the mean scores between these two groups were significantly different (*p* = 0.000). Contrastingly, those whose 1st-degree members were diagnosed with any genetic disorder found fewer “Benefits to action” (4.48 ± 0.69) than those who did not report any genetic disorder among their 1st degree relatives (4.56 ± 0.59). *t*-test showed that mean scores for these two groups were significantly (*p* = 0.041) different (*t* = 2.045; df = 1,280; 95%CI = 0.00 to 0.17). Additionally, the former reported less “Barriers to action” (3.97 ± 0.79) than the latter (4.11 ± 0.76). The *t*-test showed that mean scores for these two groups were noticeably (*p* = 0.008) different (*t* = 2.644; df = 1,280; 95%CI = 0.04 to 0.24). No other significant difference was noted in any other theme (Supplementary Tables 6A,B).

### 3.10 Variation in mean of themes when parents were close relatives

Social acceptance for PMS was highest among the responders whose parents had no relationship (4.47 ± 0.78), while it was lower if they were 1st-degree cousins (4.32 ± 0.91), and it was lowest if the parents belonged to the same tribe (4.26 ± 0.91). The results were found to vary significantly at (*p* < 0.05) (Supplementary Table 7).

### 3.11 Difference in mean of responders when they are obliged to consanguineous marriages

Participants who were forced into sanguineous marriages reported the highest susceptibility (3.87 ± 0.80) and seriousness (3.76 ± 0.97) but showed the lowest mean for Self-efficacy (4.11 ± 0.96) and social acceptance for PMS (3.77 ± 1.13). All these means were significantly (all *p* < 0.01) different than those who were not forced into sanguineous marriages or whose response was “Do not know.” Also, the participants who were forced into blood marriages accepted that Benefits and cues to action toward PMS will be beneficial (*p* < 0.01) and it can help prevent at-risk marriages. These participants had the highest mean for benefits to action (4.60 ± 0.62) and for cues to action (4.66 ± 0.65). However, an unexpected outcome was noticed, when the participants who were forced into at-risk marriages reported the lowest mean for Barriers to action (3.84 ± 0.93) as compared to others. This could be attributed to the low number of participants (*n* = 122) who accepted that they were forced into sanguineous marriages (Supplementary Tables 8A,B).

### 3.12 Multiple regression analysis to explore the predictability of themes

Multiple regression analysis was conducted to examine the effect of other themes on the perceived seriousness of PMS. ANOVA results show that the model is a good fit (*R* = 16.5%; *F* = 50.020 at *p* = 0.000), which means the data is correlated. Susceptibility had the highest predictive value (*β* = 0.312; *t* = 9.595 at *p* < 0.001) followed by Benefits to action (*β* = 0.295; *t* = 7.075 at *p* < 0.001) and Self-efficacy (*β* = 0.092; *t* = 2.760 at *p* < 0.05). All three themes showed positive predictability, which means that participants’ seriousness to PMS could be greater if they were more susceptible or saw more benefits to action.

Benefits to action had the highest positive prediction value (*β* = 0.235; *t* = 7.354 at *p* < 0.001) for susceptibility, followed by seriousness (*β* = 0.184; *t* = 9.595 at *p* < 0.001) and Cues to action (*β* = 0.112; *t* = 4.735 at *p* < 0.001). In contrast, social acceptance (*β* = −0.044; *t* = −19.85 at *p* < 0.05) and Barriers to action (*β* = −0.100; *t* = −4.375 at *p* < 0.000) had negative predictive values. This may imply that those who are serious about PMS have higher chances of taking action to prevent the at-risk marriages. Social acceptance had the lowest prediction for benefits to action (*β* = 0.095; *t* = 5.412 at *p* < 0.001), which may imply that responders who have the courage to take action might be considered socially unacceptable. Social acceptance had the lowest prediction for benefits to action (*β* = 0.095; *t* = 5.412 at *p* < 0.001), which may imply that responders who have the courage to take action might be considered socially unacceptable.

Multiple regression outcomes illustrate that 39.1% of the variation in barriers to action is due to susceptibility, self-efficacy, social acceptance, and benefits to action. Benefits to action had the highest positive predictive value (*β* = 0.220; *t* = 6.104 at *p* < 0.001), followed by Self-efficacy (*β* = 0.183; *t* = 6.47 at *p* < 0.001). This may be considered to imply that responders who saw benefits to action toward PMS and had better self-efficacy saw fewer barriers to their action. Perceived susceptibility toward PMS showed negative prediction for barriers to action (*β* = −0.125; *t* = −4.375 at *p* < 0.001).

Benefits to action had the highest positive predictive value (*β* = 0.382; *t* = 11.418 at *p* < 0.001) for cues to action, followed by Self-efficacy (*β* = 0.178; *t* = 6.58 at *p* < 0.001). This may be considered to imply that responders who saw benefits to action toward PMS and had better self-efficacy saw fewer barriers to their action. Susceptibility and social acceptance also showed significant positive predictive values, which may mean that the responders with higher susceptibility and better social acceptance will find the cues to action to benefit them to avoid at-risk marriages.

Multiple logistic regression indicates that 2.7% of the variation in barriers to action is attributed to the predictors mentioned in Table 3, *F* (14,1508) = 4.050, *p* = 0.000. The outcomes indicate that traditions of marriage among relatives are a significant barrier to rejecting consanguineous marriages. Although our study focused on understanding how unmarried individuals perceive the risks and benefits of PMS using the HBM constructs, there are certainly other cultural beliefs and influences, as well as possible misinformation, that could impact individuals’ decisions about PMS. Cultural beliefs and norms surrounding consanguineous marriages, the perceived importance of maintaining family traditions, and the impact of social pressures are all potential factors that may influence individuals’ attitudes toward PMS. Additionally, misinformation or lack of awareness about the benefits and implications of PMS within certain communities may contribute to the variation in decisions related to PMS.

**Table 3:** The outcomes of multinomial regression.

Predictors of barriers to action	Std. coefficients (*β*)	*t*-test value	*p*
Gender	−0.84	−3.197	0.001
Education	−0.073	−2.582	0.010
Have any of your 1st-degree family members (parents or siblings) been diagnosed with a genetic disease?	0.067	2.567	0.010
Does the traditions of your family, tribe, or society oblige you to marry from your relatives?	0.114	4.421	0.000

## 4 Discussion

In highly consanguineous populations, PMS becomes an essential method for primary prevention. PMS can encourage unmarried couples to reconsider marriage and learn about reproductive health. In the Arabian Peninsula, consanguineous marriages and tribal marriages have caused genetic disorders to be common (27, 29). According to the previous reports the rate of consanguinity ranges from 25 to 60% in the gulf countries (27, 30, 31), our findings align with these reports where the consanguinity rate is 35.41% among the participants’ parents; 22.01% of them are first degree relatives. The prevalence in our study is lower than the previously reported rates, this may be due the efforts of the government in increasing the awareness toward consanguinity and the benefits of avoiding at-risk marriages.

Previous research shows that health beliefs are a key factor in determining an individual’s health behaviors and outcomes (32). Our analysis indicates that the perceived severity of these diseases is positively linked to susceptibility, the benefits of taking action, and self-efficacy. Similarly, perceived susceptibility is positively associated with the benefits of taking action, severity, and cues to action, while social acceptance and barriers to action have negative predictive values. We also considered demographic variables, which have been shown to impact preventive behavior and participation in PMS (33). Our study confirms that certain demographic variables significantly influence the intentions and behaviors of couples.

In some studies, demographic factors affect whether premarital couples undergo screening (34, 35). Only a small portion of participation decisions is explained by gender, age, ethnicity, residence, profession, education, and monthly income. Additionally, studies have shown that PMS awareness, knowledge, and attitudes affect whether premarital couples attend screening.

### 4.1 Influence of age on health beliefs

The outcomes of our study reveal that youngest age group 18–25 assume themselves to be at higher risk of having a child with genetic condition if they avoid PMS, this implies that this age group possesses sufficient information and understands the fatality of the CM. This was in contrast to the previous studies which stated that older age groups show more knowledge and seriousness than the younger groups (35, 36). However, the perceived seriousness of the diseases included in the PMS was highest among older age group 34–41 followed by 26–33 age group (Figure 2) and it aligns with the previous reports.

### 4.2 Influence of gender on health beliefs

Our results reveal that females are more susceptible than males (Supplementary Tables 1A,B), which aligns with the results reported by Alhowiti et al. (19). This may be due to the fact that women are more concerned about chronic illnesses that can impact both themselves and their offspring’s well-being (19). Regarding other HBM factors such as seriousness, benefits to action, cues to action, self-efficacy, and social acceptance for PMS, women displayed more prominent actions than men, except for barriers to action. Another reason for this could be the higher number of female participants in our study.

### 4.3 Influence of marital status on the themes

Married individuals scored higher in all constructs except cues to action when compared to those who had never been married. This finding is consistent with another study conducted by Al-Shroby et al. in 2021 (21). The higher mean across all the themes may be because they had already taken the PMS before their first marriage, however, their low mean for Cues to action for safe marriage was not understood. It might be because they did not understand how they can practice safe marriage, or they assume that these cues may not facilitate their action against at-risk marriages.

### 4.4 Influence of education

The impact of education on various aspects is significant. For instance, those with a diploma tend to have a lower mean score for the “Barriers to action,” while postgraduates enjoy significantly higher social acceptability than diploma holders (37). Similarly, we found diploma holders to have significantly lower barriers to action as compared to those with higher education, while individuals with a postgraduate degree enjoy the highest social acceptability. It may be because the higher level of education allows the respondents to comprehend the risk of CM and hence enhance their perception of social acceptability. So, it may be deduced that better education widens the scopes accepting the outcomes of PMS and will consequently play a vital role in avoiding at-risk CM.

### 4.5 Influence of family income

The present study reveals that individuals with a family income lower than 10 k demonstrated a lower degree of social acceptability compared to their counterparts with a family income above 10 k. Notably, our findings align with the results of a prior investigation conducted by Binshihon et al. (26). However, no statistically significant differences were observed in any other themes assessed in our study.

### 4.6 Impact of previous diagnoses of genetic diseases or any type of disability

Research indicates that individuals diagnosed with genetic diseases may be more susceptible to experiencing PMS than those without such a diagnosis (38, 39). It is a strong predictor of perceived susceptibility among participants and is consistent with existing literature (21). However, responders with genetic disorders may not benefit as much from cues to action as those without a diagnosis. No significant differences in other constructs were observed, and no significant differences were found between responders with disabilities and those without.

### 4.7 Impact of family history of genetic disorders or disability

Our study suggests that participants with a family history of genetic disorders may have a higher susceptibility and a lower perception of benefits to action. This factor is a good predictor of participants’ susceptibility, reason being the presence of a familial medical background, which may heighten the significance of a disease and does not alter one’s perceived ability to prevent the disease (40). Also, these participants reported lower “Barriers to action” as real-life consequences of at-risk marriage in their own family may have influenced their perception toward these barriers.

### 4.8 Influence of blood relationship of participant’s parents

Participants who agreed that their parents were not closely related had the lowest mean for susceptibility, but their perceived seriousness was same as those whose parents were 1st degree cousins. This may imply that the former may have the perception that they are at low risk of having a child with genetic condition since their parents are not related. However, their seriousness toward the diseases included in PMS might be due to their knowledge of genetic inheritance of disorders. Our research also revealed that participants whose parents were unrelated had the highest level of social acceptance toward PMS, compared to those who were 1st-degree cousins or had to marry within the same tribe. This is understandable as these participants’ parents were unrelated and it may be assumed that they are already avoiding at-risk marriages and hence practice of safe marriage easier and acceptable.

### 4.9 Influence of obligation to marry within the tribe

The study revealed an interesting finding that participants who were forced into CM reported the highest levels of susceptibility and severity toward PMS. However, they experienced low self-efficacy and social acceptance. While they had high perceived benefits and cues to action, they reported the lowest mean for barriers to action. This could be because of the low number of participants (*n* = 122) who acknowledged being forced into CM.

### 4.10 Recommendation

According to the study, a significant number of participants, specifically females (65.6%), would reconsider their decision to marry if premarital testing revealed incompatibility. However, a considerable percentage of participants (50% males and 35% females) would still proceed with an unsafe marriage despite the incompatible results. This highlights the importance of analyzing the factors influencing health-related beliefs to promote healthier practices. The study identified several factors, such as a history of genetic disease, family history of genetic disease or disability, and traditions of marrying close relatives as strong predictors of perceived susceptibility. Higher education and family member history of genetic disease or disability were linked to lower perceived barriers to action. Furthermore, traditions of marrying close relatives were identified as a significant barrier to rejecting consanguineous marriages. The study found that gender and the presence of genetic disorders among family members had an impact on the perceived benefits to action, while gender and family member history of genetic disease were predictors of cues to action. Gender and traditions of marrying close relatives were predictors of self-efficacy, whereas higher education and female gender were associated with higher social acceptance. On the other hand, a family history of genetic disease or disability was linked to negative social acceptance.

The importance of spreading awareness about the benefits of PMS for genetic disease prevention was highlighted in the study. Overcoming perceived obstacles can be achieved through education and social support. Cultivating positive social attitudes toward PMS is crucial, particularly among men and those with limited education. Personalized strategies should be created to address various socio-demographic groups’ diverse requirements and concerns.

## 5 Limitation

The participants included in this study were mainly females (1370) as compared to males (85), so the interpretation and generalizability of the outcomes are only fit for female population. The disproportionate participation in this study could be due to the survey being conducted by a woman’s university. Some reports have also stated that males are less likely to participate in a survey study than females. Additionally, we acknowledge that the cross-sectional design of our study limits the establishment of causal relationships. Therefore, future research should consider longitudinal or qualitative approaches to gain a deeper understanding of the specific cultural beliefs, misinformation, and social factors that influence decisions about PMS. Furthermore, the sample may not be representative of the entire unmarried population. While this study sheds light on the importance of addressing barriers and promoting positive social acceptance to enhance the effectiveness of PMS, further research is needed to explore these cultural beliefs, misinformation, and other external factors that play a crucial role in shaping individuals’ decisions regarding PMS. More research is necessary to examine the effectiveness of interventions aimed at increasing PMS adoption and reducing consanguineous marriages.

## 6 Conclusion

The presented study provides valuable insights into the health beliefs and attitudes surrounding PMS among unmarried individuals. By identifying and addressing barriers and promoting positive social acceptance, PMS can potentially contribute greatly to preventing genetic diseases that cause disability and promoting safe marriage practices.

## Data availability statement

The original contributions presented in the study are included in the article/Supplementary material, further inquiries can be directed to the corresponding author.

## Ethics statement

The studies involving humans were approved by Institutional Review Board, Princess Nourah Bint Abdulrahman University. The studies were conducted in accordance with the local legislation and institutional requirements. The participants provided their written informed consent to participate in this study.

## Author contributions

RA: Conceptualization, Funding acquisition, Project administration, Resources, Software, Supervision, Validation, Writing – original draft, Writing – review & editing. AA: Conceptualization, Investigation, Methodology, Validation, Writing – original draft. MA: Conceptualization, Investigation, Methodology, Validation, Writing – original draft. HA: Data curation, Investigation, Writing – original draft. SA: Data curation, Investigation, Writing – original draft. RK: Conceptualization, Data curation, Formal analysis, Investigation, Methodology, Software, Validation, Visualization, Writing – original draft, Writing – review & editing.
