# Supplementary material for: Health beliefs of unmarried adult Saudi individuals toward safe marriage and the role of premarital screening in avoiding consanguinity: a nationwide cross-sectional study
Source: Front Public Health. 2024 Jun 19;12:1379326. doi: 10.3389/fpubh.2024.1379326 (PMC11219822; doi:10.3389/fpubh.2024.1379326)
Supplement: Supplementary file 1 [file Table_1.DOCX]

**S-Table 1a: shows the mean score of the themes for all the participants wrt to the age group among males.**

|  | Male | | | | | | | | | |
| --- | --- | --- | --- | --- | --- | --- | --- | --- | --- | --- |
|  | Age Groups | | | | | | | | | |
|  | 18-25 | | 26-33 | | 34-41 | | 42-49 | | Total | |
|  | Mean | Standard Deviation | Mean | Standard Deviation | Mean | Standard Deviation | Mean | Standard Deviation | Mean | Standard Deviation |
| Perceived Susceptibility | 3.34 | .76 | 3.48 | .78 | 3.58 | .65 | 3.40 | 1.21 | 3.42 | .76 |
| Perceived Seriousness | 3.36 | .82 | 3.38 | .80 | 3.61 | 1.22 | 3.81 | 1.38 | 3.42 | .88 |
| Benefits to action | 4.17 | .96 | 4.38 | .73 | 4.42 | .68 | 4.30 | .74 | 4.27 | .84 |
| Barriers to action | 2.33 | .90 | 2.07 | .77 | 2.09 | .78 | 2.07 | .78 | 2.20 | .84 |
| Cues to action | 4.00 | .96 | 3.94 | 1.08 | 3.73 | .94 | 4.17 | 1.11 | 3.95 | .99 |
| Self-efficacy | 4.04 | 1.03 | 4.25 | .97 | 4.18 | 1.09 | 4.58 | .32 | 4.15 | .99 |
| Social acceptance | 3.92 | 1.14 | 4.38 | 1.00 | 4.58 | .63 | 3.75 | .57 | 4.15 | 1.05 |

**S-Table 1b: shows the mean score of the themes for all the participants wrt to the age group among females.**

|  | Female | | | | | | | | | |
| --- | --- | --- | --- | --- | --- | --- | --- | --- | --- | --- |
|  | Age Groups | | | | | | | | | |
|  | 18-25 | | 26-33 | | 34-41 | | 42-49 | | Total | |
|  | Mean | Standard Deviation | Mean | Standard Deviation | Mean | Standard Deviation | Mean | Standard Deviation | Mean | Standard Deviation |
| Perceived Susceptibility | 3.60 | .66 | 3.49 | .82 | 3.49 | .93 | 3.49 | 1.31 | 3.59 | .69 |
| Perceived Seriousness | 3.47 | .90 | 3.56 | .94 | 3.81 | 1.05 | 3.34 | .94 | 3.49 | .91 |
| Benefits to action | 4.55 | .58 | 4.53 | .66 | 4.57 | .70 | 4.33 | 1.21 | 4.55 | .60 |
| Barriers to action | 1.91 | .76 | 1.89 | .82 | 1.95 | .89 | 1.74 | 1.04 | 1.91 | .77 |
| Cues to action | 4.40 | .76 | 4.47 | .70 | 4.20 | .97 | 4.24 | 1.25 | 4.40 | .77 |
| Self-efficacy | 4.34 | .76 | 4.48 | .69 | 4.42 | .69 | 3.85 | 1.24 | 4.35 | .76 |
| Social acceptance | 4.36 | .85 | 4.64 | .62 | 4.56 | .69 | 4.00 | 1.22 | 4.39 | .84 |

**S-Table 2a: *Difference in the means of the themes for Previously married and unmarried participants***

| **Group Statistics** | | | | | |
| --- | --- | --- | --- | --- | --- |
|  | Have you ever been married? | N | Mean | Std. Deviation | Std. Error Mean |
| Suceptibility | No | 1481 | 3.5739 | .68977 | .01792 |
|  | Yes | 41 | 3.6829 | 1.00894 | .15757 |
| Seriousness | No | 1481 | 3.4762 | .89964 | .02338 |
|  | Yes | 41 | 3.6829 | 1.16058 | .18125 |
| Benefits_to_action | No | 1481 | 4.5282 | .61935 | .01609 |
|  | Yes | 41 | 4.6049 | .70780 | .11054 |
| BarR | No | 1481 | 4.0672 | .77405 | .02011 |
|  | Yes | 41 | 4.1951 | .84016 | .13121 |
| Cues_to_action | No | 1481 | 4.3727 | .79054 | .02054 |
|  | Yes | 41 | 4.3008 | .95686 | .14944 |
| Self_efficacy | No | 1481 | 4.3360 | .77430 | .02012 |
|  | Yes | 41 | 4.5122 | .77852 | .12158 |
| Social acceptance | No | 1481 | 4.3639 | .85527 | .02222 |
|  | Yes | 41 | 4.6179 | .77661 | .12129 |

**S-Table 2b: *Difference in the means of the themes for Previously married and unmarried participants***

| **Independent Samples Test** | | | | | | | | | | |
| --- | --- | --- | --- | --- | --- | --- | --- | --- | --- | --- |
|  | | Levene's Test for Equality of Variances | | t-test for Equality of Means | | | | | | |
|  |  | F | Sig. | t | df | Sig. (2-tailed) | Mean Difference | Std. Error Difference | 95% Confidence Interval of the Difference | |
|  |  |  |  |  |  |  |  |  | Lower | Upper |
| Susceptibility | Equal variances assumed | 12.769 | .000 | -.983 | 1520 | .326 | -.10899 | .11083 | -.32639 | .10841 |
|  | Equal variances not assumed |  |  | -.687 | 41.042 | .496 | -.10899 | .15859 | -.42925 | .21127 |
| Seriousness | Equal variances assumed | 7.765 | .005 | -1.439 | 1520 | .150 | -.20673 | .14367 | -.48854 | .07509 |
|  | Equal variances not assumed |  |  | -1.131 | 41.342 | .264 | -.20673 | .18275 | -.57571 | .16226 |
| Benefits_to_action | Equal variances assumed | .021 | .884 | -.779 | 1520 | .436 | -.07672 | .09845 | -.26983 | .11639 |
|  | Equal variances not assumed |  |  | -.687 | 41.713 | .496 | -.07672 | .11170 | -.30220 | .14875 |
| BarR | Equal variances assumed | .020 | .888 | -1.041 | 1520 | .298 | -.12789 | .12284 | -.36883 | .11306 |
|  | Equal variances not assumed |  |  | -.963 | 41.901 | .341 | -.12789 | .13274 | -.39580 | .14002 |
| Cues_to_action | Equal variances assumed | 2.525 | .112 | .571 | 1520 | .568 | .07191 | .12592 | -.17509 | .31891 |
|  | Equal variances not assumed |  |  | .477 | 41.526 | .636 | .07191 | .15084 | -.23261 | .37642 |
| Self_efficacy | Equal variances assumed | .623 | .430 | -1.437 | 1520 | .151 | -.17616 | .12261 | -.41665 | .06433 |
|  | Equal variances not assumed |  |  | -1.429 | 42.220 | .160 | -.17616 | .12324 | -.42483 | .07251 |
| Social_acceptance | Equal variances assumed | 6.118 | .013 | -1.880 | 1520 | .060 | -.25394 | .13509 | -.51893 | .01105 |
|  | Equal variances not assumed |  |  | -2.059 | 42.730 | .046 | -.25394 | .12330 | -.50266 | -.00523 |

**S-Table 3a: *Difference in the means of responders with different education status.***

| **Multiple Comparisons** | | | | | | | |
| --- | --- | --- | --- | --- | --- | --- | --- |
| Tukey HSD | | | | | | | |
| Dependent Variable | (I) Education_Level | (J) Education_Level | Mean Difference (I-J) | Std. Error | Sig. | 95% Confidence Interval | |
|  |  |  |  |  |  | Lower Bound | Upper Bound |
| BarR | High school and below | Diploma | .29269^*^ | .08541 | .004 | .0730 | .5124 |
|  |  | University | -.07055 | .04550 | .408 | -.1876 | .0465 |
|  |  | Postgraduate | -.16040 | .08756 | .259 | -.3856 | .0648 |
|  | Diploma | High school and below | -.29269^*^ | .08541 | .004 | -.5124 | -.0730 |
|  |  | University | -.36324^*^ | .08085 | .000 | -.5712 | -.1553 |
|  |  | Postgraduate | -.45309^*^ | .11015 | .000 | -.7364 | -.1698 |
|  | University | High school and below | .07055 | .04550 | .408 | -.0465 | .1876 |
|  |  | Diploma | .36324^*^ | .08085 | .000 | .1553 | .5712 |
|  |  | Postgraduate | -.08986 | .08311 | .701 | -.3036 | .1239 |
|  | Postgraduate | High school and below | .16040 | .08756 | .259 | -.0648 | .3856 |
|  |  | Diploma | .45309^*^ | .11015 | .000 | .1698 | .7364 |
|  |  | University | .08986 | .08311 | .701 | -.1239 | .3036 |
| Social_acceptance | High school and below | Diploma | .13210 | .09424 | .498 | -.1103 | .3745 |
|  |  | University | -.08409 | .05020 | .337 | -.2132 | .0450 |
|  |  | Postgraduate | -.30723^*^ | .09661 | .008 | -.5557 | -.0588 |
|  | Diploma | High school and below | -.13210 | .09424 | .498 | -.3745 | .1103 |
|  |  | University | -.21619 | .08921 | .073 | -.4456 | .0132 |
|  |  | Postgraduate | -.43933^*^ | .12154 | .002 | -.7519 | -.1267 |
|  | University | High school and below | .08409 | .05020 | .337 | -.0450 | .2132 |
|  |  | Diploma | .21619 | .08921 | .073 | -.0132 | .4456 |
|  |  | Postgraduate | -.22314 | .09171 | .071 | -.4590 | .0127 |
|  | Postgraduate | High school and below | .30723^*^ | .09661 | .008 | .0588 | .5557 |
|  |  | Diploma | .43933^*^ | .12154 | .002 | .1267 | .7519 |
|  |  | University | .22314 | .09171 | .071 | -.0127 | .4590 |
| *. The mean difference is significant at the 0.05 level. | | | | | | | |

**S-Table 3b: *Difference in the means of responders with different education status***

| **Descriptives** | | | | | | | | | |
| --- | --- | --- | --- | --- | --- | --- | --- | --- | --- |
|  | | N | Mean | Std. Deviation | Std. Error | 95% Confidence Interval for Mean | | Minimum | Maximum |
|  |  |  |  |  |  | Lower Bound | Upper Bound |  |  |
| BarR | High school and below | 420 | 4.0381 | .74228 | .03622 | 3.9669 | 4.1093 | 1.00 | 5.00 |
|  | Diploma | 101 | 3.7454 | .99110 | .09862 | 3.5497 | 3.9411 | 1.00 | 5.00 |
|  | University | 906 | 4.1086 | .76319 | .02536 | 4.0589 | 4.1584 | 1.00 | 5.00 |
|  | Postgraduate | 95 | 4.1985 | .69528 | .07133 | 4.0569 | 4.3401 | 2.14 | 5.00 |
|  | Total | 1522 | 4.0707 | .77589 | .01989 | 4.0317 | 4.1097 | 1.00 | 5.00 |
| Social_acceptance | High school and below | 420 | 4.3103 | .89178 | .04351 | 4.2248 | 4.3959 | 1.00 | 5.00 |
|  | Diploma | 101 | 4.1782 | .95866 | .09539 | 3.9890 | 4.3675 | 1.00 | 5.00 |
|  | University | 906 | 4.3944 | .83828 | .02785 | 4.3397 | 4.4491 | 1.00 | 5.00 |
|  | Postgraduate | 95 | 4.6175 | .62450 | .06407 | 4.4903 | 4.7448 | 2.00 | 5.00 |
|  | Total | 1522 | 4.3708 | .85401 | .02189 | 4.3278 | 4.4137 | 1.00 | 5.00 |

**S-Table 4: *Difference in the mean of the themes wrt to the income level***

| **Independent Samples Test** | | | | | | | | | | |
| --- | --- | --- | --- | --- | --- | --- | --- | --- | --- | --- |
|  | | Levene's Test for Equality of Variances | | t-test for Equality of Means | | | | | | |
|  |  | F | Sig. | t | df | Sig. (2-tailed) | Mean Difference | Std. Error Difference | 95% Confidence Interval of the Difference | |
|  |  |  |  |  |  |  |  |  | Lower | Upper |
| Social_acceptance | Equal variances assumed | 7.873 | .005 | -2.063 | 1520 | .039 | -.09495 | .04602 | -.18522 | -.00467 |
|  | Equal variances not assumed |  |  | -2.002 | 977.214 | .046 | -.09495 | .04743 | -.18803 | -.00187 |

**S-Table 5: *Difference in the mean score of responders diagnosed with genetic disease***

| **Independent Samples Test** | | | | | | | | | | |
| --- | --- | --- | --- | --- | --- | --- | --- | --- | --- | --- |
|  | | Levene's Test for Equality of Variances | | t-test for Equality of Means | | | | | | |
|  |  | F | Sig. | t | df | Sig. (2-tailed) | Mean Difference | Std. Error Difference | 95% Confidence Interval of the Difference | |
|  |  |  |  |  |  |  |  |  | Lower | Upper |
| Suceptibility | Equal variances assumed | 2.350 | .125 | -5.632 | 1520 | .000 | -.38967 | .06919 | -.52538 | -.25395 |
|  | Equal variances not assumed |  |  | -5.201 | 120.971 | .000 | -.38967 | .07492 | -.53799 | -.24134 |
| Cues_to_action | Equal variances assumed | 2.788 | .095 | 2.016 | 1520 | .044 | .15991 | .07931 | .00435 | .31547 |
|  | Equal variances not assumed |  |  | 1.859 | 120.911 | .065 | .15991 | .08603 | -.01041 | .33023 |

**S-Table 6a: *Variation in mean score of themes if any 1^st^ degree relative had genetic anomalies***

| **Independent Samples Test** | | | | | | | | | | |
| --- | --- | --- | --- | --- | --- | --- | --- | --- | --- | --- |
|  | | Levene's Test for Equality of Variances | | t-test for Equality of Means | | | | | | |
|  |  | F | Sig. | t | df | Sig. (2-tailed) | Mean Difference | Std. Error Difference | 95% Confidence Interval of the Difference | |
|  |  |  |  |  |  |  |  |  | Lower | Upper |
| Suceptibility | Equal variances assumed | .629 | .428 | -6.538 | 1280 | .000 | -.31501 | .04818 | -.40954 | -.22048 |
|  | Equal variances not assumed |  |  | -6.326 | 391.530 | .000 | -.31501 | .04980 | -.41292 | -.21711 |
| Benefits_to_action | Equal variances assumed | 5.919 | .015 | 2.045 | 1280 | .041 | .08612 | .04212 | .00348 | .16876 |
|  | Equal variances not assumed |  |  | 1.872 | 368.206 | .062 | .08612 | .04601 | -.00436 | .17661 |
| BarR | Equal variances assumed | 1.435 | .231 | 2.644 | 1280 | .008 | .13973 | .05286 | .03603 | .24344 |
|  | Equal variances not assumed |  |  | 2.581 | 395.719 | .010 | .13973 | .05415 | .03329 | .24618 |

**S-Table 6b: *Variation in mean score of themes if any 1^st^ degree relative had genetic anomalies***

| **Group Statistics** | | | | | |
| --- | --- | --- | --- | --- | --- |
| Have any of your 1st-degree family members (parents or siblings)been diagnosed with a genetic disease? | | N | Mean | Std. Deviation | Std. Error Mean |
| Suceptibility | No | 1019 | 3.4972 | .68840 | .02157 |
|  | Yes | 263 | 3.8122 | .72796 | .04489 |
| Benefits_to_action | No | 1019 | 4.5637 | .58827 | .01843 |
|  | Yes | 263 | 4.4776 | .68378 | .04216 |
| BarR | No | 1019 | 4.1126 | .75771 | .02374 |
|  | Yes | 263 | 3.9728 | .78923 | .04867 |

**S-Table 7: *Variation in mean score of themes when parents were close relatives***

| **Multiple Comparisons** | | | | | | | |
| --- | --- | --- | --- | --- | --- | --- | --- |
| Tukey HSD | | | | | | | |
| Dependent Variable | | | Mean Difference (I-J) | Std. Error | Sig. | 95% Confidence Interval | |
|  |  |  |  |  |  | Lower Bound | Upper Bound |
| Social_acceptance | No relationship | 1st degree cousin | .15482^*^ | .05772 | .037 | .0064 | .3033 |
|  |  | 2nd degree cousin | .10586 | .06869 | .413 | -.0708 | .2825 |
|  |  | related to the same tribe | .21229^*^ | .05607 | .001 | .0681 | .3565 |
| *. The mean difference is significant at the 0.05 level. | | | | | | | |

**S-Table 8a: Difference in mean of responders when they are obliged to consanguineous marriages**

| **Multiple Comparisons** | | | | | | | |
| --- | --- | --- | --- | --- | --- | --- | --- |
| Tukey HSD | | | | | | | |
| Dependent Variable | | | Mean Difference (I-J) | Std. Error | Sig. | 95% Confidence Interval | |
|  |  |  |  |  |  | Lower Bound | Upper Bound |
| Susceptibility | No | Yes | -.33873^*^ | .06577 | .000 | -.4930 | -.1844 |
|  |  | Don't Know | -.15204^*^ | .06046 | .032 | -.2939 | -.0102 |
| Seriousness | Yes | No | .29157^*^ | .08575 | .002 | .0904 | .4927 |
|  |  | Don't Know | .39800^*^ | .11073 | .001 | .1382 | .6578 |
| Barriers to action | No | Yes | .27940^*^ | .07303 | .000 | .1081 | .4507 |
|  |  | Don't Know | .24394^*^ | .06713 | .001 | .0865 | .4014 |
| Cues to action | Yes | No | .31856^*^ | .07502 | .000 | .1426 | .4946 |
|  |  | Don't Know | .29159^*^ | .09688 | .007 | .0643 | .5189 |
| Self-efficacy | No | Yes | .26779^*^ | .07301 | .001 | .0965 | .4391 |
|  |  | Don't Know | .21021^*^ | .06712 | .005 | .0528 | .3677 |
| Social acceptance | No | Yes | .70642^*^ | .07785 | .000 | .5238 | .8891 |
|  |  | Don't Know | .54087^*^ | .07156 | .000 | .3730 | .7088 |
| *. The mean difference is significant at the 0.05 level. | | | | | | | |

**S-Table 8b: Difference in mean of responders when they are obliged to consanguineous marriages**

| **Descriptives** | | | | | | | | | |
| --- | --- | --- | --- | --- | --- | --- | --- | --- | --- |
|  | | N | Mean | Std. Deviation | Std. Error | 95% Confidence Interval for Mean | | Minimum | Maximum |
|  |  |  |  |  |  | Lower Bound | Upper Bound |  |  |
| Susceptibility | No | 1253 | 3.5350 | .68923 | .01947 | 3.4968 | 3.5732 | 1.00 | 5.00 |
|  | Yes | 122 | 3.8738 | .79719 | .07217 | 3.7309 | 4.0167 | 1.00 | 5.00 |
|  | Don't Know | 147 | 3.6871 | .63551 | .05242 | 3.5835 | 3.7907 | 1.40 | 5.00 |
|  | Total | 1522 | 3.5769 | .70003 | .01794 | 3.5417 | 3.6121 | 1.00 | 5.00 |
| Seriousness | No | 1253 | 3.4687 | .89860 | .02539 | 3.4189 | 3.5185 | 1.00 | 5.00 |
|  | Yes | 122 | 3.7602 | .96605 | .08746 | 3.5871 | 3.9334 | 1.00 | 5.00 |
|  | Don't Know | 147 | 3.3622 | .89814 | .07408 | 3.2158 | 3.5086 | 1.00 | 5.00 |
|  | Total | 1522 | 3.4818 | .90779 | .02327 | 3.4361 | 3.5274 | 1.00 | 5.00 |
| Benefits_to_action | No | 1253 | 4.5312 | .61136 | .01727 | 4.4973 | 4.5651 | 1.00 | 5.00 |
|  | Yes | 122 | 4.5934 | .62241 | .05635 | 4.4819 | 4.7050 | 1.20 | 5.00 |
|  | Don't Know | 147 | 4.4694 | .70289 | .05797 | 4.3548 | 4.5840 | 1.00 | 5.00 |
|  | Total | 1522 | 4.5302 | .62176 | .01594 | 4.4990 | 4.5615 | 1.00 | 5.00 |
| BarR | No | 1253 | 4.1166 | .74991 | .02119 | 4.0751 | 4.1582 | 1.00 | 5.00 |
|  | Yes | 122 | 3.8372 | .92606 | .08384 | 3.6713 | 4.0032 | 1.00 | 5.00 |
|  | Don't Know | 147 | 3.8727 | .79700 | .06574 | 3.7428 | 4.0026 | 2.00 | 5.00 |
|  | Total | 1522 | 4.0707 | .77589 | .01989 | 4.0317 | 4.1097 | 1.00 | 5.00 |
| Cues_to_action | No | 1253 | 4.3426 | .80412 | .02272 | 4.2981 | 4.3872 | 1.00 | 5.00 |
|  | Yes | 122 | 4.6612 | .64707 | .05858 | 4.5452 | 4.7772 | 1.33 | 5.00 |
|  | Don't Know | 147 | 4.3696 | .78623 | .06485 | 4.2415 | 4.4978 | 1.00 | 5.00 |
|  | Total | 1522 | 4.3708 | .79518 | .02038 | 4.3308 | 4.4108 | 1.00 | 5.00 |
| Self_efficacy | No | 1253 | 4.3825 | .73888 | .02087 | 4.3416 | 4.4235 | 1.00 | 5.00 |
|  | Yes | 122 | 4.1148 | .96283 | .08717 | 3.9422 | 4.2873 | 1.00 | 5.00 |
|  | Don't Know | 147 | 4.1723 | .84612 | .06979 | 4.0344 | 4.3103 | 1.00 | 5.00 |
|  | Total | 1522 | 4.3408 | .77468 | .01986 | 4.3018 | 4.3797 | 1.00 | 5.00 |
| Social_acceptance | No | 1253 | 4.4796 | .76188 | .02152 | 4.4374 | 4.5219 | 1.00 | 5.00 |
|  | Yes | 122 | 3.7732 | 1.12755 | .10208 | 3.5711 | 3.9753 | 1.00 | 5.00 |
|  | Don't Know | 147 | 3.9388 | .98930 | .08160 | 3.7775 | 4.1000 | 1.00 | 5.00 |
|  | Total | 1522 | 4.3708 | .85401 | .02189 | 4.3278 | 4.4137 | 1.00 | 5.00 |

**S-Table 9: Gender wise description of responses (in %) to the questions (n=1522). P shows the significance value for difference.**

| **Themes** | **Sentences for Themes (P value)** | **1** | **2** | **3** | **4** | **5** |
| --- | --- | --- | --- | --- | --- | --- |
|  |  | **Male/female (% within Gender)** | | | | |
|  |  | **Strongly Disagree** | **Disagree** | **Neutral** | **Agree** | **Strongly Agree** |
| **Perceived Susceptibility** | Consanguineous marriage increases the risk of having a child with a genetic disease. (P=0.003) | 6.5/2.5 | 2.2/2.9 | 23.7/13.0 | 28.0/26.4 | 39.8/55.2 |
|  | If one of my family members diagnosed with a genetic disease (or a carrier), this makes me at higher risk of having a child with genetic disease. (P=0.016) | 10.8/3.8 | 6.5/6.2 | 28.0/24.4 | 26.9/31.3 | 28.0/34.3 |
|  | Thinking that the possibility of transmission of the genetic disease to my children is high. (P=0.370) | 39.8/46.9 | 26.9/23.4 | 24.7/18.8 | 6.5/6.2 | 2.2/4.8 |
|  | I care to know if I am at risk of getting a child with genetic disease. (P=0.156) | 6.5/4.1 | 4.3/1.9 | 8.6/5.7 | 14.0/11.3 | 66.7/77.0 |
|  | I worry a lot about having a child with a genetic disease. (P=0.787) | 17.2/19.5 | 11.8/13.6 | 26.9/21.4 | 12.9/13.8 | 31.2/31.6 |
| **Perceived Seriousness** | I believe that all diseases covered by premarital screening test are severe health problem that could lead to disability in children. (P=0.395) | 7.5/6.1 | 9.7/9.6 | 28.0/20.2 | 16.1/20.1 | 38.7/44.1 |
|  | If my child diagnosed with SCD (one of the diseases included in premarital screening test), he/she could suffer from fetal complications including hemorrhagic stroke, supraventricular tachycardia, and DVT. (P=0.317) | 4.3/2.9 | 4.3/4.7 | 43.0/34.9 | 23.7/23.1 | 24.7/34.5 |
|  | If my child diagnosed with SCD (one of the diseases included in premarital screening test), he/she could have intellectual and physical disabilities. (P=0.366) | 15.1/15.5 | 8.6/14.9 | 46.2/37.4 | 14.0/14.8 | 16.1/17.4 |
|  | Sickle cell anemia or Thalassemia (diseases included in premarital screening test) are genetic diseases that can’t be treated and might lead to lifelong disability. (P=0.408) | 6.5/6.4 | 11.8/11.2 | 43.0/33.9 | 15.1/20.4 | 23.7/28.0 |
|  | If my child diagnosed with Thalassemia (one of the diseases included in premarital screening test), he/she could suffer from complications including endocrine system, heart, liver, and spleen problems. (P=0.938) | 3.2/3.2 | 3.2/5.3 | 47.3/47.1 | 21.5/20.7 | 24.7/23.7 |
| **Benefits of action** | The premarital screening test will inform my decision to practice safe marriage. (P=0.001) | 3.2/1.0 | 3.2/1.3 | 6.5/2.9 | 17.2/8.8 | 69.9/86.0 |
|  | A genetic disease can be prevented if carrier partners are discovered before marriage. (P=0.000) | 5.4/1.5 | 1.1/1.3 | 11.8/4.5 | 20.4/12.7 | 61.3/79.9 |
|  | I trust the results of the premarital screening test regarding the risk of having a child with a genetic disease. (P=0.715) | 3.2/2.2 | 4.3/3.3 | 16.1/12.3 | 22.6/25.0 | 53.8/57.3 |
|  | If PMS showed that there is a risk of having a child with genetic disease, I will take the decision of canceling the marriage. (P=0.013) | 5.4/2.1 | 5.4/3.3 | 15.1/12.6 | 24.7/16.4 | 49.5/65.6 |
|  | My decision to practice safe marriage, following a PMS result, will prevent me from the psychological and economic burden of having a child with genetic disease. (P=0.011) | 2.2/1.5 | 6.5/2.6 | 8.6/8.5 | 23.7/13.9 | 59.1/73.6 |
| **Barriers to action** | I am afraid that if I stopped the marriage that could weaken the relationships within a tribe. (P=0.001) | 44.1/63.1 | 17.2/15.0 | 21.5/11.5 | 11.8/5.7 | 5.4/4.6 |
|  | I am afraid that if I stopped the marriage my chances of getting a suitable spouse will be affected. (P=0.000) | 39.8/66.8 | 16.1/13.4 | 24.7/9.7 | 14.0/5.8 | 5.4/4.4 |
|  | I am afraid that if I cancel the marriage my family members' (sisters/brothers) chances of getting a suitable spouse will be affected. (P=0.000) | 52.7/72.3 | 17.2/13.0 | 21.5/8.5 | 6.5/3.6 | 2.2/2.6 |
|  | I am not convinced that premarital screening test result is accurate and making the decision based on it will not protect my future kids from getting genetic disease or disability. (P=0.921) | 50.5/53.4 | 21.5/18.6 | 17.2/16.1 | 6.5/8.0 | 4.3/3.8 |
|  | Prior emotional bond with my partner could hinder me from canceling the marriage. (P=0.596) | 23.7/29.2 | 14.0/15.3 | 33.3/26.1 | 15.1/15.2 | 14.0/14.2 |
|  | Pressure from the family could hinder me from canceling the marriage. (P=0.278) | 47.3/55.5 | 19.4/14.1 | 19.4/14.1 | 9.7/9.8 | 4.3/6.5 |
|  | Financial status could hinder me from canceling the marriage. (P=0.006) | 38.7/58.4 | 22.6/13.5 | 21.5/15.9 | 8.6/5.7 | 8.6/6.6 |
| **Cues to action** | An educational consultation before and after the PMS, could limit my intention in the incompatible marriage. (P=0.343) | 9.7/6.4 | 4.3/4.5 | 26.9/22.0 | 26.9/24.9 | 32.3/42.1 |
|  | Attending educational courses in high schools which raise health awareness on the importance of premarital screening and spreading awareness about safe marriage in the community will increase the safe marriage behavior. (P=0.000) | 8.6/2.4 | 10.8/2.4 | 18.3/9.0 | 20.4/18.7 | 41.9/67.4 |
|  | Receiving SMS text messages about the seriousness of the diseases included in the PMS test will help me, my family and my community to practice safe marriage. (P=0.016) | 9.7/4.8 | 8.6/6.2 | 22.6/14.3 | 17.2/19.2 | 41.9/55.5 |
|  | Having a supporting family or friends will assist me to practice safe marriage. (P=0.040) | 2.2/1.7 | 3.2/1.1 | 10.8/8.2 | 23.7/15.2 | 60.2/73.8 |
| **Self-efficacy** | I believe I am capable of canceling the marriage if the result is incompatible. (P=0.000) | 9.7/2.5 | 2.2/2.7 | 12.9/11.6 | 25.8/19.1 | 49.5/64.1 |
|  | It is up to me to take the right decision regarding safe marriage. (P=0.103) | 5.4/2.4 | 2.2/1.7 | 11.8/8.1 | 20.4/15.3 | 60.2/72.4 |
|  | I can practice safe marriage even if I don’t get support from my family. (P=0.865) | 3.2/3.8 | 4.3/6.6 | 20.4/17.9 | 18.3/16.6 | 53.8/55.1 |
| **Social acceptability** | My decision of safe marriage does not contradict with the customs and traditions of my family, tribe, or community. (P=0.023) | 6.5/2.4 | 6.5/3.5 | 16.1/11.5 | 17.2/15.5 | 53.8/67.0 |
|  | My family will accept and support my decision of safe marriage based on the result of the premarital examination. (P=0.000) | 6.5/1.3 | 0.0/2.1 | 15.1/10.1 | 18.3/16.1 | 60.2/70.5 |
|  | My tribe and community will accept and support my decision of safe marriage based on the result of the premarital examination. (P=0.608) | 5.4/2.7 | 4.3/5.0 | 17.2/18.2 | 18.3/15.9 | 54.8/58.2 |
